# SASCI/SCTSSA joint consensus statement and guidelines on transcatheter aortic valve implantation (TAVI ) in South Africa

**DOI:** 10.5830/CVJA-2016-092

**Published:** 2016

**Authors:** Jacques Scherman, Hellmuth Weich

**Affiliations:** Chris Barnard Division of Cardiothoracic Surgery, University of Cape Town, South Africa; Division of Cardiology, Tygerberg Hospital and Stellenbosch University, Cape Town, South Africa

## Introduction

The South African Heart Association (SA Heart) together with two of its special-interest groups, the South African Society of Cardiovascular Intervention (SASCI) and the Society of Cardiothoracic Surgeons in South Africa (SCTSSA), represent the scientific, educational and professional interests of South African cardiac specialists, with a combined membership of over 200 members. These two interest groups exclusively represent practicing cardiologists and cardiothoracic surgeons in South Africa. SASCI and SCTSSA are dedicated to maintaining the highest standards of specialist practice and the highest quality of patient care. As a result, SASCI and SCTSSA seek to serve as a knowledge resource for patients and funders in matters related to new technology used in the cardiac interventional and surgical disciplines.

The introduction of new technology is a constant in modern medicine. While authorities in the United States of America (USA) and European Union, such as the Food and Drug Administration (FDA) and Conformité Européene (CE), provide regulatory clearance on safety and effectiveness, practicing medical practitioners require scientific evidence on net health outcomes before offering new procedures to their patients. In addition, to meet clinical expectations of practicing specialists, new technology must stay consistent with fundamental medical and surgical principles.

Transcatheter aortic valve implantation (TAVI) is considered a feasible technique, which may be used as an alternative to standard surgical aortic valve replacement in selected cases. The procedure is performed on the beating heart without the need for a sternotomy or cardiopulmonary bypass. There are currently two devices available in South Africa that are CE-marked and approved by the FDA. The procedure may be performed via the transfemoral, transsubclavian and transapical approaches or via a mini-sternotomy (transaortic approach).

SA Heart and the respective boards of the SASCI and SCTSSA by consensus hereby adopt the TAVI procedure for aortic stenosis in line with the principles of evidence-based medicine after considering the most recent published evidence and the various multinational society position statements and guidelines concerning TAVI.

This consensus guideline considers all the literature reviewed, including the 2014 American Heart Association/American College of Cardiology guideline for the management of patients with valvular heart disease, the 2012 European Society of Cardiology/European Association for Cardiothoracic Surgery guidelines on the management of valvular heart disease, and the updated standardised endpoint definitions for TAVI [as per the Valve Academic Research Consortium-2 (VARC-2) consensus document].^1^-^3^

## Consensus guidelines on transcatheter aorticvalve implantation (TAVI)

Members of the SA Heart Association, SASCI and SCTSSA with experience in the technique and knowledge of the TAVI literature have agreed to the following consensus statement:

## Requirements and structure of the multidisciplinary heart team

The performance of TAVI, ab initio, should be restricted to a limited number of high-volume centres, which have both cardiology and cardiac surgery departments on site, with expertise in structural heart disease intervention and high-risk valvular surgery. Interventional cardiologists should be experienced in catheter-based valvular interventions and peripheral access using large devices. Cardiac surgeons should be experienced in valve surgery and the management of complex cases. It is recommended that all TAVI teams aim to perform more than 10 implants per year.
TAVI should currently be reserved for patients who, after evaluation by a multidisciplinary heart team (MDT) are found to have a risk/benefit ratio favouring TAVI rather than open-heart surgery. The heart team should at least include a cardiologist, cardiac surgeon and imaging specialist. Its composition is however dynamic and can also include a cardiac anaesthetist, geriatrician and neurologist as well as other members as the MDT sees fit.
Patients should be screened into a TAVI programme by a MDT (as defined above) and not by an individual specialist.
Formal training of the implanting team should include:
  didactic theoretical training
  simulator training where available
  a visit to an experienced centre to observe TAVI cases
  support for the initial cases at any site by a proctor until the proctor has certified the centre to be independent.

## Patient selection/mandatory prerequisites

Proof of severe symptomatic aortic valve stenosis.
Patient evaluation by a MDT.

## Indications for TAVI

TAVI is recommended in patients who are, according to the MDT heart team, considered to be unsuitable for conventional surgery because of severe co-morbidities. These include:
  Possible procedure-specific impediment, for example:
    porcelain aorta or severely atherosclerotic aorta
    hostile chest
    patent coronary artery bypass grafts crossing the midline and/or adherent to the posterior table of the sterum
OR
  Frailty. In the absence of validated frailty scores, this remains the opinion of an experienced physician. We recommend that it is the opinion of at least two physicians of which one should be a cardiac surgeon experienced in aortic valve replacement surgery
OR
  Major organ compromise of two or more organ systems. Examples include:
    cardiac: severe left or right ventricular dysfunction, severe pulmonary hypertension
    pulmonary dysfunction (FEV1 or DLCO2 < 50% predicted)
    central nervous system dysfunction (dementia, Alzheimer’s disease, Parkinson’s disease)
    gastro-intestinal dysfunction (Chron’s disease, ulcerative colitis)
    liver cirrhosis, variceal bleeding.
TAVI is recommended in patients who are, according to the MDT, considered to be at high risk for conventional surgery. In line with other guidelines, the evaluation of surgical risk should rely on the clinical judgement of an MDT rather than quantitative risk scores as these have not been well validated in this population. These risk scores may be used in addition, with cut-off values of an STS (Society of Thoracic Surgeons) risk score > 4 or a log EuroSCORE > 20 recommended. It must be emphasised that risk scores should not be used in isolation to determine whether a patient qualifies to undergo a TAVI procedure. Growing evidence supports the efficacy of TAVI in ‘intermediate-risk group’ patients.^4^ The final recommendation therefore remains with the MDT.

## Contra-indications

Absence of an MDT heart team and no cardiac surgery on site.
Patients whose life expectancy is less than one year.
Clinical improvement in quality of life after TAVI limited by co-morbidities. This may be especially relevant if the indication for TAVI is major organ compromise, as outlined above.
Anatomical factors
  inadequate annulus size
  active endocarditis
  inadequate access site.
Significant other valve lesions or coronary artery disease that requires additional valve or coronary artery bypass surgery.
Relative contra-indications
  left ventricular ejection fraction (LVEF) < 20%
  haemodynamic instability.

## Establishing a TAVI programme

The centre should be sufficiently equipped to perform transcatheter procedures safely.^1^-^3^
Minimum infrastructure requirements include:
  The ability to set up an MDT (as outlined above)
  Immediate availability of transthoracic and transoesophageal echocardiography.
  Availability of a dedicated cardiac catheterisation laboratory or hybrid theatre [a theatre with mobile fluoroscopy (‘C’-arm) screening facilities is generally not appropriate for TAVI procedures].
  Computed tomography (CT) scanning facilities.
  Immediate availability of perfusion services in case emergency cardiopulmonary bypass (extracorporeal circulation) becomes necessary.
  On-site availability of a surgical recovery area and intensive care with staff experienced in looking after patients following surgical aortic valve replacement.
  Facilities for immediate renal support if necessary.
  Immediate access to vascular surgery and interventional radiology to deal with peripheral vascular complications.
The above requirements will mean that this procedure should only be performed in a unit currently carrying out surgical aortic valve replacement.
